# Scar burden is an independent and incremental predictor of cardiac resynchronisation therapy response

**DOI:** 10.1136/openhrt-2019-001067

**Published:** 2019-07-05

**Authors:** Serge C Harb, Saleem Toro, Jennifer A Bullen, Nancy A Obuchowski, Bo Xu, Kevin M Trulock, Niraj Varma, John Rickard, Richard Grimm, Brian Griffin, Scott D Flamm, Deborah H Kwon

**Affiliations:** 1 Heart and Vascular Institute, Cleveland Clinic, Cleveland, Ohio, USA; 2 Quantitative Health Sciences, Cleveland Clinic, Cleveland, Ohio, USA

**Keywords:** cardiac MRI, cardiac resynchronisation therapy response, incremental value

## Abstract

**Objective:**

Determine the prognostic impact of scar quantification (scar %) by cardiac magnetic resonance (CMR) in predicting heart failure admission, death and left ventricular (LV) function improvement following cardiac resynchronisation therapy (CRT), after controlling for the presence of left bundle branch block (LBBB), QRS duration (QRSd) and LV lead tip location and polarity.

**Methods:**

Consecutive patients who underwent CMR between 2002 and 2014 followed by CRT were included. The primary endpoint was death or heart failure admission. The secondary endpoint was change in ejection fraction (EF) ≥3 months after CRT. Cox proportional hazards, linear regression models and change in the area under the receiver operating characteristic curve (AUC) were used.

**Results:**

A total of 84 patients were included (63% male, 51% with ischaemic cardiomyopathy). After adjusting for clinical factors, presence of LBBB and QRSd and LV lead tip location and polarity, greater scar % remained associated with a higher risk for clinical events (HR=1.06; 95% CI 1.02 to 1.10; p<0.001) and a smaller improvement in EF (slope: −0.61%; 95% CI −0.93% to 0.29%; p<0.001). When adding scar % to QRSd and LBBB, there was significant improvement in predicting clinical events at 3 years (AUC increased to 0.831 from 0.638; p=0.027) and EF increase ≥10% (AUC 0.869 from 0.662; p=0.007).

**Conclusion:**

Scar quantification by CMR has an incremental value in predicting response to CRT, in terms of heart failure admission, death and EF improvement, independent of the presence of LBBB, QRSd, LV lead tip location and polarity.

**Key questions**

**What is already known about this subject?**

- Approximately one-third of patients who receive cardiac resynchronisation therapy (CRT) do not benefit and better identification of patients who will respond is crucial. Scar quantification by cardiac magnetic resonance (CMR) has been postulated to predict clinical response to CRT; however, its independent and incremental value to currently adopted selection criteria (presence of left bundle branch block and QRS complex duration), and when taking into account left ventricular (LV) lead characteristics and the type of cardiomyopathy, has not been evaluated.

**What does this study add?**

- Scar quantification by CMR appears to independently predict clinical events and LV functional improvement, in both ischaemic and non-ischaemic cardiomyopathy, even when accounting for clinical, electrocardiographic and LV lead characteristics. It adds incremental value to currently adopted selection criteria for the prediction of CRT response.

**How might this impact on clinical practice?**

- Scar quantification by CMR prior to CRT implantation may allow better identification of patients who would respond to this therapy.

## Introduction

Cardiac resynchronisation therapy (CRT) is an established therapeutic option for select heart failure patients with reduced ejection fraction (EF) and ECG evidence of dyssynchrony.^1 2^ A wealth of evidence from randomised controlled trials has supported two main predictors of benefit from CRT^3–5^: a wide QRS complex and the presence of left bundle branch block (LBBB) morphology. This has driven major society and government-sponsored guidelines^6 7^ to adopt these two variables as the main selection criteria for CRT implantation. Despite improvements with this therapy, approximately one-third of patients who receive a CRT do not benefit^8 9^ and are deemed ‘non-responders’, and further deterioration in left ventricular (LV) function can also occur in a small subset of patients.^10^ Current guidelines advocating patient selection based solely on QRS morphology and duration are clearly limited. In an attempt to improve the selection process, a multitude of variables have been studied. Among these, myocardial scar, by cardiac magnetic resonance (CMR)^11–14^ or nuclear imaging,^15–17^ has shown great potential in identifying non-responders. Prior studies have used varied definition of ‘response’, and the response rate has differed when defined as 6 min hall walk distances and improvement in quality of life scores compared with more objective endpoints such as heart failure hospitalisations and death.^18^ However, these prior studies have not assessed the incremental benefit of defining myocardial scar compared with current clinical criteria (presence of LBBB and QRS duration (QRSD) ≥120 ms) and have not evaluated the prognostic impact on survival and heart failure admissions.

We sought to assess whether scar quantification by CMR is independently and incrementally predictive heart failure hospitalisation and death after CRT implantation when accounting for clinical data, the presence of LBBB and QRSd, and LV lead tip position in relation to scar^19^ and lead polarity.^20 21^

## Methods

### Patient population

All consecutive patients who underwent CMR testing at our institution (Cleveland Clinic, Cleveland, Ohio, USA) between January 2002 and June 2014, and had subsequent CRT implantation were initially included. Patients who underwent CRT-P (CRT-Pacemaker) without defibrillator (CRT-D) and those with significant time delay between the CMR scan and CRT implantation (more than 1-year for ischaemic cardiomyopathy (ICM) and more than 2 years for non-ischaemic cardiomyopathy (NICM)) were excluded. Baseline clinical characteristics were determined by chart review. Ischaemic cardiomyopathy was defined as presence of severe coronary disease on cardiac catheterisation, prior coronary revascularisation (coronary bypass surgery or percutaneous intervention) and by its characteristic scar pattern on CMR.^22^

### Cardiovascular MRI

**Figure 1:**
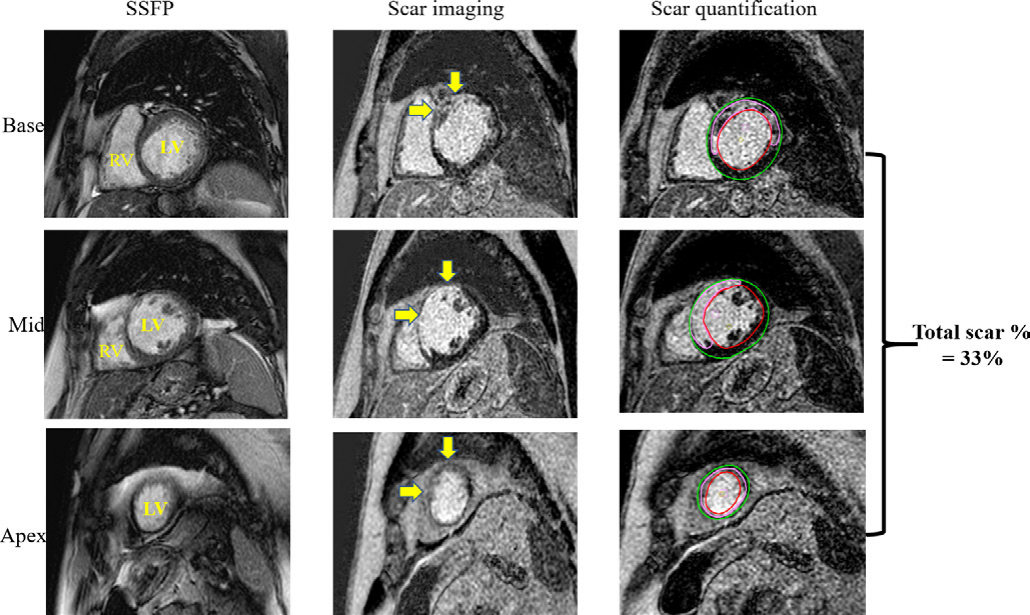
Cardiac MRI and scar quantification. Example of cardiac magnetic resonance short axis stack acquisition, scar imaging and scar quantification from a patient with prior left anterior descending artery infarction and resultant ischaemic cardiomyopathy. For presentation purposes, only three slices (one at the base, one at the mid cavity and one at the apex) are shown. The yellow arrows point to the scar. Scar quantification is performed by first defining the endocardial (in red) and epicardial (in green) contours. Scar is shown here in pink. For accurate calculation of total scar %, the entire LV stack needs to be contoured from the atrioventricular junction (mitral annulus) to the apex. LV, left ventricle; RV, right ventricle; SSFP, steady state free precession.

The CMR scan was performed an average of 87 days before CRT implantation. All studies were obtained on dedicated CMR scanners (Achieva 1.5 T XR and Ingenia 3.0 T; Philips Medical Systems, Best, The Netherlands). For assessment of global cardiac function, steady-state free precession short axis LV stack slices were acquired (sequential slices of 8 mm thickness from the mitral annulus to the apex with 2 mm interslice gap). Delayed hyperenhancement (DHE) images were obtained 10 min after intravenous injection of 0.2 mmol/kg of Gadolinium meglumine (Dotarem). Scar was considered present if seen on two orthogonal views. The short axis DHE images were then analysed using commercially available software (cvi42; Circle Cardiovascular Imaging, Calgary, Alberta, Canada). Endocardial and epicardial borders were manually delineated. Scar was then defined using a threshold of >2 SD compared with user-defined viable myocardium for ICM and >6 SD for NICM, as these thresholds have been shown to correlate most accurately with histological fibrosis.^23 24^ The scar percentage (total scar %) was then automatically determined as percentage of total myocardium (figure 1).

In order to explore whether regional variations in scar % had an impact on CRT response, we divided the myocardium into four walls (septal, anterior, inferior and lateral) comprised of the following American Heart Association (AHA) segments: septum (AHA segments 2, 3, 8, 9, 14), anterior (AHA segments 1, 7 and 13), inferior (AHA segments 4, 10, 15) and lateral (AHA segments 5, 6, 11, 12 and 16).

### LV lead tip location and type

The LV lead tip location was determined using post-CRT implantation postero-anterior and lateral chest radiographs.^25^ The lateral projection was used to categorise the lead tip position in the anterior, lateral or inferior wall. Then, correlation with MRI regional scar distribution, as described above, was performed to determine whether the lead tip was in a normal versus scarred myocardial wall. The LV lead type (unipolar vs multipolar) was determined by review of the procedural note. Both bipolar and quadripolar LV leads were grouped as multipolar.

### Endpoints

Response to CRT was assessed both clinically and echocardiographically.

#### Clinical endpoint

The primary endpoint was defined as time from CRT to death or heart failure admission. Events were determined from medical records by two independent reviewers (SCH and ST) and the first event was included in the analysis. The final censor date was 14 October 2017. The mean follow-up duration was 3.7 years.

#### Echocardiographic endpoint

Transthoracic echocardiography (TTE) was performed on all patients prior to CRT implantation (on average 50 days prior). The EF was measured by biplane method of disks (modified Simpson’s rule) or estimated visually, in case the former was technically challenging. To assess response to CRT, we measured the EF on a TTE performed ≥90 days after CRT implantation (on average, the repeat TTE was performed 2.2 years after CRT) to allow sufficient time for the therapy to have effect. ‘Responders’ were defined as those whose EF increased by at least 10% after CRT, as lower thresholds of change in EF by the biplane method can be related to test–retest variability.^26 27^

### Statistical analysis

A Cox proportional hazards model was used for the composite clinical endpoint and a linear regression model was used for the EF endpoint. Total scar % was included as the primary predictor of interest. The following variables were added as covariates: clinical—age, gender, diabetes mellitus and hypertension; electrocardiographic—LBBB and QRSd (in ms); and LV lead characteristics—lead tip location (in scar vs normal myocardium) and polarity (unipolar vs multipolar). In order to assess whether the impact of scar on patient outcome is different for ICM and NICM patients, an interaction term was added.

To be useful clinically, scar quantification by CMR needs to be not only of independent value but also of incremental value to currently adopted CRT selection criteria (presence of LBBB and QRS width). For this purpose, the incremental improvement in overall predictive accuracy due to scar was assessed by the change in the area under the receiver operating characteristic (ROC) curve. Clinical events were determined at both 1 and 3 years in accordance to published clinical trial data.^28^ All analyses were performed using R V.3.4.1. Time-to-event analyses made use of the ‘survival’ package.

## Results

A total of 111 patients were initially included. Patients receiving CRT-P (n=4), ICM patients who received CRT more than 1 year after CMR (n=16), and NICM patients who received CRT more than 2 years after CMR (n=7) were all excluded. The final sample consisted of 84 patients: 43 ICM and 41 NICM. Table 1 presents the baseline characteristics of the overall population, and by type of cardiomyopathy (ICM vs NICM).

**Table 1:** Baseline characteristics of the overall population and by cardiomyopathy type

	All patients (n=84)	ICM (n=43)	NICM (n=41)
Clinical characteristics			
Age (mean±SD, in years)	62±12	66±9	57±12
Female (%)	37	35	39
Diabetes mellitus (%)	21	28	15
Hypertension (%)	40	28	54
Hyperlipidaemia (%)	42	44	39
Atrial fibrillation* (%)	29	35	22
NYHA Class III/IV (%)	90	91	90
ECG pre-CRT			
Presence of LBBB (%)	50	40	61
QRS duration (mean±SD in ms)	151±24	147±23	154±24
Echo characteristics			
EF pre-CRT (mean %±SD)	23±8	21±8	26±8
EF ≥3 months post CRT (mean %±SD)	34±14	28±13	40±13
CMR characteristics			
LVEF (mean %±SD)	24±9	20±8	28±9
Total scar % (mean %±SD)	15±17	26±16	5±10

*Includes permanent, persistent and paroxysmal atrial fibrillation.

EF, ejection fraction;ICM, ischaemic cardiomyopathy;LBBB, left bundle branch block;LVEF, left ventricular ejection fraction;NICM, non-ischaemic cardiomyopathy;NYHA, New York Heart Association;QRS, QRS complex duration in ms.

The lead tip location and type were available in 67 patients. The LV lead tip was located in the lateral wall in 32 (48%) patients and in the inferior wall in 33 (49%) patients. Two patients had the LV lead tip in the anterior wall. In relation to regional scar by MRI, 43 patients had their lead tip in scarred myocardium and 24 had their lead tip in normal myocardium. The lead was multipolar in 44 (66%) cases (bipolar in 34 and quadripolar in 10) and unipolar in 23 (34%) cases.

### Clinical endpoint

**Figure 2:**
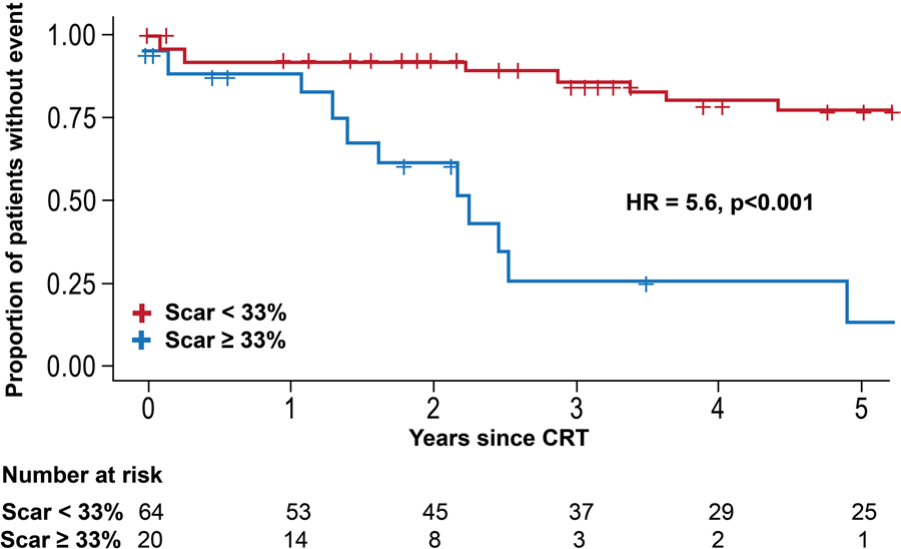
Kaplan-Meier estimates of the survivor function for the primary endpoint (composite of death or heart failure admission) when adopting a scar threshold of 33%. Patients with higher burden of scar had more events compared with those with lower scar burden. In this figure, a threshold of 33% is selected for presentation purposes. Patients with scar ≥33% had significantly more events compared with those with <33% (HR: 5.6; 95% CI 2.4 to 12.7, p<0.001). CRT, cardiac resynchronisation therapy.

**Figure 3:**
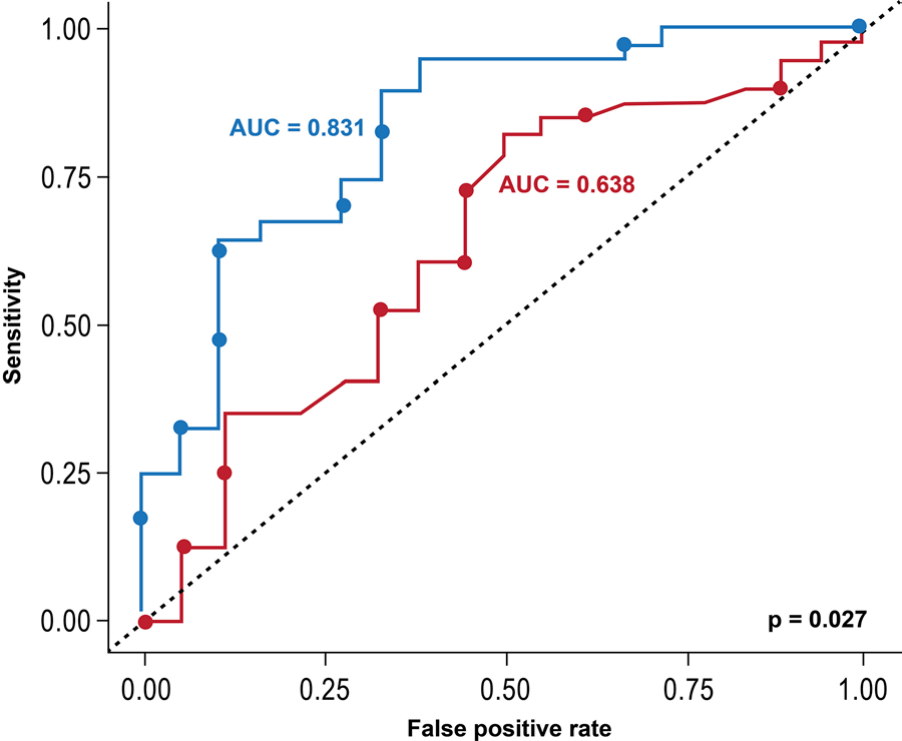
Receiver-operating characteristics (ROCs) curves for distinguishing patients who were event-free for the primary composite outcome 3 years after cardiac resynchronisation therapy the primary outcome was defined as death or heart failure admission. In predicting the occurrence of this composite outcome at 3 years, the red line represents the ROC based on the presence of left bundle branch block and QRS duration. The blue line represents the ROC curve after addition of total scar %. There was a significant improvement in the predictive accuracy of the model (95% CI for the difference in area under the receiver operating characteristic curve (AUC) 0.02 to 0.36; p=0.027).

A total of 29 patients experienced an adverse event during follow-up (heart failure in 19 and death in 10). Total scar % was significantly higher in patients who had an event compared with those who were event free for the primary endpoint (22% vs 12%, p=0.02). After adjusting for the presence of LBBB and QRSd, clinical, and LV lead characteristics, older age and greater levels of scar were associated with a higher risk of the composite clinical endpoint (HR per 1% increase in scar: 1.06, 95% CI for HR 1.02 to 1.10, p<0.001). On the other hand, the presence of LBBB was associated with a lower risk of clinical events after CRT (table 2). Figure 2 shows the Kaplan-Meier survival estimates for the primary composite endpoint when adopting, for presentation purposes, a scar threshold of 33%. Patients with scar ≥33% had significantly more events compared with those with less scar burden (HR: 5.6; 95% CI 2.4 to 12.7, p<0.001). No significant difference was observed between NICM and ICM patients with respect to this effect (interaction p value: 0.429). In addition, there was a significant improvement in the predictive accuracy, with the area under the ROC increasing from 0.638 to 0.831, when total scar % was added to the predictive model (95% CI for the difference in area under the receiver operating characteristic curve (AUC) 0.02 to 0.36; p=0.027) (figure 3). We then sought to determine the impact of scar location on clinical outcome. The LV myocardium was divided into four walls (anterior, septum, lateral and inferior) based on AHA segments classification, and the scar % in each of these walls was calculated as the average of the segments included. When multivariable analysis was performed, substituting consecutively total scar % by site specific scar %, an increase in the scar burden in each of the four walls was associated with worse outcomes (table 3).

**Table 2:** Univariable and multivariable associations with the post cardiac resynchronisation therapy (CRT) clinical endpoints (heart failure admission or death)

	Univariable		Multivariable	
	HR with 95% CI	P value	HR with 95% CI	P value
Age	1.04 (1.00 to 1.08)	0.035	1.07 (1.01 to 1.13)	0.017
Female	0.76 (0.36 to 1.61)	0.469	0.97 (0.43 to 2.22)	0.945
Total scar (per 1% increase)	1.05 (1.03 to 1.07)	<0.001	1.06 (1.02 to 1.10)	0.003
LBBB	0.55 (0.26 to 1.17)	0.119	0.29 (0.09 to 0.94)	0.039
QRS width	0.99 (0.98 to 1.01)	0.427	1.01 (0.98 to 1.04)	0.600
Diabetes	0.88 (0.37 to 2.09)	0.770	0.63 (0.23 to 1.73)	0.367
Hypertension	0.79 (0.36 to 1.74)	0.561	1.32 (0.53 to 3.32)	0.549
LV lead tip in scar	2.26 (0.84 to 6.08)	0.106	0.62 (0.16 to 2.37)	0.486
Unipolar lead	2.76 (1.24 to 6.14)	0.013	1.06 (0.34 to 3.28)	0.918

On multivariable analysis, only increasing age, increasing scar burden and absence of LBBB were associated with lack of clinical response to CRT (heart failure admission or death).

LBBB, left bundle branch block; LV, left ventricle; QRS, QRS complex duration in ms.

**Table 3:** Estimates of the effect of location-specific scar % on the clinical and echocardiographic endpoints

	Adjusted* estimate of HR							
	Septal scar†		Anterior scar‡		Lateral scar§		Inferior scar¶	
A: clinical endpoint	Estimate	P value	Estimate	P value	Estimate	P value	Estimate	P value
Time between CRT and death or heart failure admission	1.03	0.002	1.03	<0.001	1.03	0.005	1.02	0.017

	Adjusted* estimate of slope							
	Septal scar†		Anterior scar‡		Lateral scar§		Inferior scar¶	
B: echocardiographic endpoint	Estimate	P value	Estimate	P value	Estimate	P value	Estimate	P value
Change in EF after CRT	−0.26	0.002	−0.24	<0.001	−0.20	0.044	−0.31	0.008

*Adjusted estimates are from a model where the following covariates were included as predictors (in addition to total scar %): age, gender, presence of left bundle branch block, QRS duration, diabetes mellitus and hypertension.

†Septal scar was calculated as the average of the scar per cent in AHA segments 2, 3, 8, 9 and 14.

‡Anterior scar was calculated as the average of the scar per cent in AHA segments 1, 7 and 13.

§Lateral scar was calculated as the average of the scar per cent in AHA segments 5, 6, 11, 12 and 16.

¶Inferior scar was calculated as the average of the scar per cent in AHA segments 4, 10 and 15.

AHA, American Heart Association; CRT, cardiac resynchronisation therapy; EF, ejection fraction.

### LV function

To address the effect of scar % on LV function, a secondary substudy analysis was performed on the patients who had TTE ≥3 months post CRT. This was available for review in 57 patients. LV EF increased by an average of 10% after CRT (range: −18% to 35%). Echocardiographic response was defined as an increase by ≥10% in EF, ≥3 months after CRT implantation.

**Figure 4:**
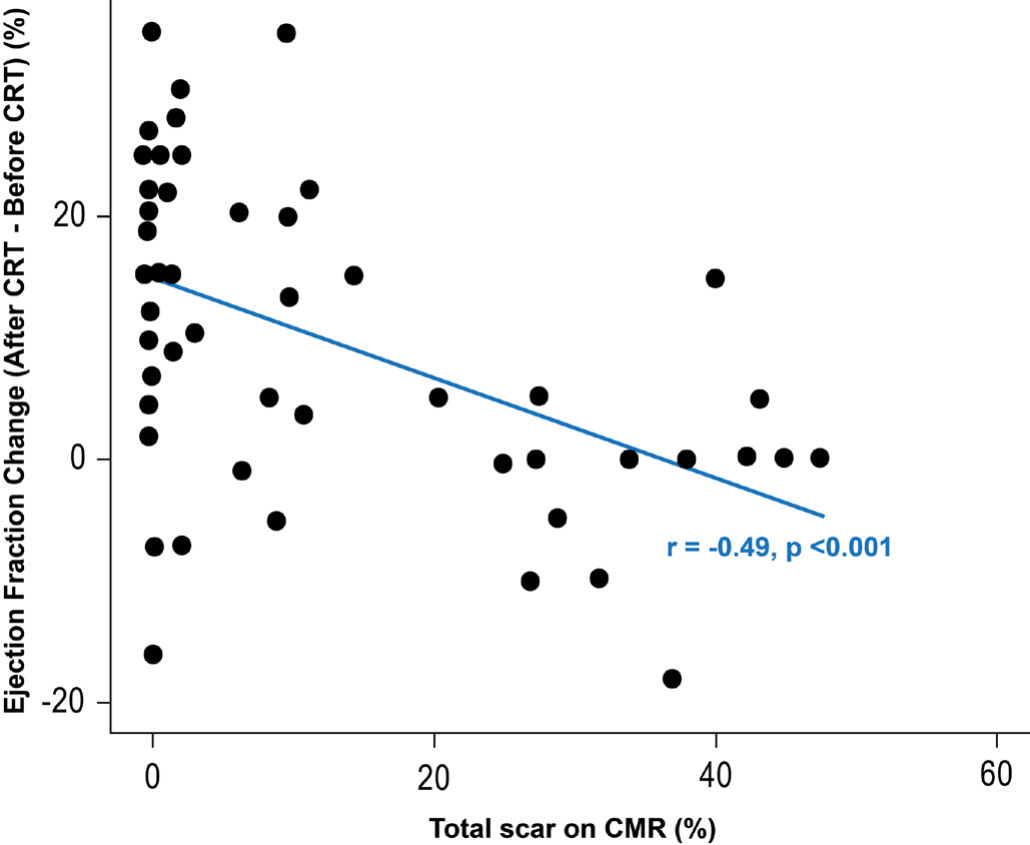
Associations between scar burden and change in ejection fraction (EF) lower scar % was associated with a larger increase in EF after cardiac resynchronisation therapy (CRT) (r=−0.49, 95% CI −0.66 to 0.26, p<0.001). Increase in EF by 10% or more (area shaded in darker grey) was considered clinically significant improvement. CMR, cardiac magnetic resonance.

**Figure 5:**
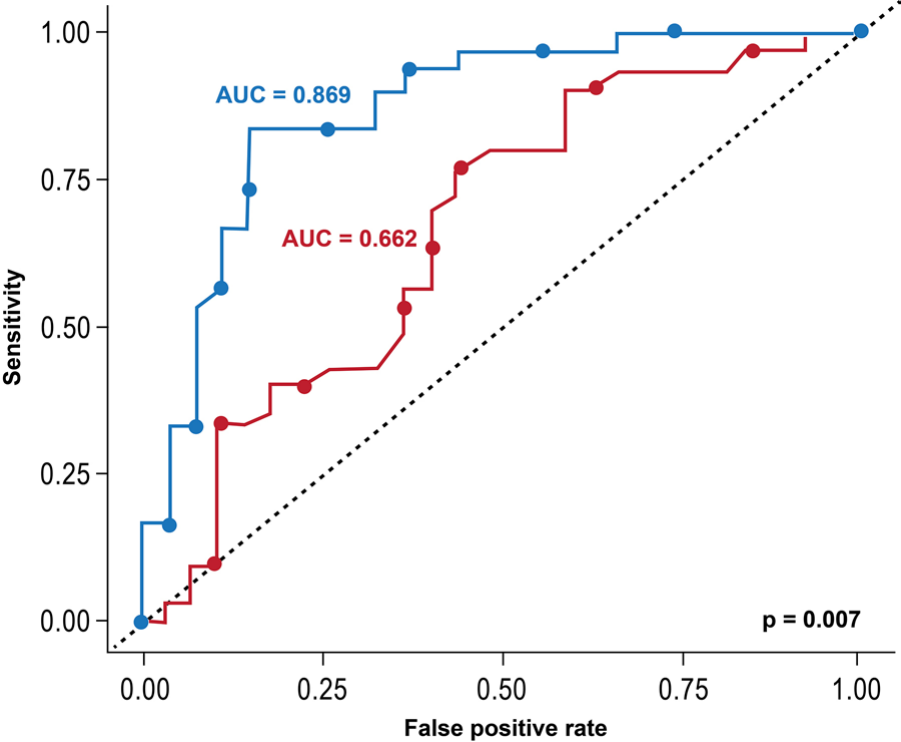
Receiver-operating characteristics (ROC) curves for distinguishing patients with significant ejection fraction (EF) improvement (≥10%) the red line represents the ROC curve for predicting EF improvement by ≥10% based on the presence of left bundle branch block and QRS duration. The blue line represents the ROC curve after adding total scar %. There was a significant improvement in the predictive accuracy of the model (95% CI for the difference in area under the receiver operating characteristic curve (AUC): 0.06, 0.36; p=0.007).

Overall, 30 patients (53%) showed significant EF improvement (ie, an increase in EF by 10% or more after CRT), while 27 (42%) did not. Figure 4 shows the change in EF by total scar %, with an inverse relationship demonstrated: the smaller the scar %, the larger the increase in EF (r: −0.49; 95% CI −0.66 to 0.26, p<0.001). On multivariable analysis, smaller levels of scar and the presence of LBBB were associated with larger increases in EF after CRT (Scar−slope: −0.61%, 95% CI −0.93% to −0.29%, p<0.001; LBBB−slope: 8.9%, 95% CI 0.4% to 17.4%, p=0.04) (table 4). The scar effect was not significantly different for NICM and ICM patients (interaction p value: 0.624). Patients who had an EF increase after CRT were at lower risk of the composite clinical endpoint (adjusted HR for 1% increase in EF=0.95; 95% CI 0.91 to 0.98, p=0.005). When the presence of LBBB and QRSd were the only predictors of response, the area under the ROC curve was 0.662. When total scar % was added as a third predictor of improvement in EF, the area under the ROC curve increased to 0.869, representing a statistically significant improvement in the predictive accuracy of the model (95% CI for the difference in AUC 0.06 to 0.36; p=0.007) (figure 5).

**Table 4:** Univariable and multivariable associations for the echocardiographic endpoint

	Univariable		Multivariable	
	Slope with 95% CI	P value	Slope with 95% CI	P value
Age	−0.3% (−0.6% to 0.0%)	0.045	−0.2% (−0.6% to 0.1%)	0.185
Female	−0.8% (−7.9% to 6.2%)	0.819	−3.1% (−10.4% to 4.2%)	0.399
Total scar (per 1% increase)	−0.4% (−0.6% to −0.2%)	<0.001	−0.6% (−0.9% to −0.3%)	<0.001
LBBB	7.4% (0.9% to 14.0%)	0.028	8.9% (0.4% to 17.4%)	0.040
QRS width	0.1% (−0.1% to 0.2%)	0.409	−0.1% (−0.3% to 0.1%)	0.390
Diabetes	−1.6% (−10.0% to 6.8%)	0.706	0.2% (−8.5% to 8.9%)	0.964
Hypertension	−0.7% (−7.7% to 6.3%)	0.851	−2.4% (−9.6% to 4.8%)	0.501
LV lead tip in scar	−4.7% (−12.5% to 3.1%)	0.229	6.1% (−2.6% to 14.8%)	0.165
Unipolar lead	−7.5% (−15.5% to 0.4%)	0.064	3.3% (−5.7% to 12.3%)	0.460

Slope is the mean change in ejection fraction difference. On multivariable analysis, only increase in scar burden and absence of LBBB were associated with lack of echocardiographic response to CRT.

LBBB, left bundle branch block; LV, left ventricle; QRS, QRS complex duration in ms.

### Sensitivity and specificity thresholds

#### Clinical endpoint

Over a follow-up time of 3 years, 21 patients experienced a primary event (heart failure in 13 and death in five). When responders were defined as those who were event-free 3 years after CRT, a scar threshold of 0% (absence of scar) had a specificity of 83% with a sensitivity of 40%, while a scar threshold of 33% had a sensitivity of 93%, with a specificity of 56%. Online supplementary table 1 presents the point and interval estimates of the sensitivity and specificity at various thresholds.

10.1136/openhrt-2019-001067.supp1 Supplementary data

#### LV function

Similarly, for the identification of echocardiographic CRT responders (EF increase by ≥10%), we found that a scar threshold of 0% provides a specificity of 81% with a sensitivity of 43%, while a scar threshold of 33% provides a sensitivity of 97% with a specificity of 26%. Online supplementary table 2 presents the point and interval estimates of the sensitivity and specificity at various thresholds.

## Discussion

While several previous studies have demonstrated the independent prognostic ability for scar % by MRI to predict clinical response to CRT,^11–14^ none of these studies have evaluated the incremental benefit of scar quantification in the prediction of heart failure and death, after controlling for the presence of LBBB and QRSd. There are four main observations from our cohort of patients with cardiomyopathy (both ischaemic and non-ischaemic) and scar quantification by CMR prior to CRT: (1) non-responders (defined clinically or echocardiographically) have higher levels of scar compared with responders; (2) scar independently predicts death or heart failure hospitalisation, and EF improvement after CRT, regardless of the type of cardiomyopathy, and even when accounting presence of LBBB and QRSd; (3) there is incremental value in incorporating scar quantification to QRSd and presence of LBBB in predicting response to CRT; (4) when considering scar location separately (septal, anterior, inferior or lateral walls), an increase in scar burden was associated with worse outcomes in any location.

Lack of CRT response portends a very poor survival, with mortality up to 50% at 4 years.^29^ Current recommendations^6 7^ relying solely on QRS morphology and width are clearly limited, and more refined criteria are needed for identification of probable non-responders. Scar assessment by CMR has shown promise based on prior reports with two small studies, of 23 and 34 patients,^11 12^ respectively, showing higher scar burden in CRT non-responders; however, both studies did not include clinical endpoints of death or heart failure. Another study of 45 patients^13^ showed that scar location, specifically in the posterolateral segments, and scar size (≥33%) are important determinants of lack of CRT response. However, this study also did not include death or heart failure as outcomes. Other studies have demonstrated that LV lead position and polarity may also impact CRT response. CMR-guided CRT placement away from scar location has been shown to result in better clinical outcomes,^19^ and multipolar leads, in comparison to unipolar ones, have been suggested to improve CRT response in part because of increased flexibility of LV lead pacing vector.^20 21^ There are also limited, though conflicting data from small studies, on whether scar location impacts CRT outcomes, with one report^11^ showing that septal scar was the main determinant, while others suggest that scar in the posterolateral region, where the CRT LV lead is typically located, is most relevant.^30 31^ In our study, when looking at site-specific scar, we found that scar in any location (anterior, inferior, lateral or septal) is associated with worse outcomes, both in terms of LV function improvement and clinical events. This likely reflects the fact that presence of scar in any wall will impede global LV remodelling and response to resynchronisation therapy, and will continue to confer a heightened risk for clinical events.

Furthermore, a subset of patients may experience worsening LV function after CRT, suggesting that CRT can result in increased harm in select patients.^10^ Therefore, there is need for improving the current patient selection criteria for CRT. In our patient cohort, the current clinical selection criteria yielded an AUC of only 0.638 for predicting heart failure and death and an AUC of 0.662 for predicting improvement in LV function. However, the findings of our study suggest that scar % may provide important independent and incremental prognostic ability to predict which patients are at high risk for ‘non-response’. We found that wen scar % was added to the clinical selection criteria of QRSd and LBBB, the AUC for predicting heart failure hospitalisation and death increased to 0.831 from 0.638; p=0.027, and the AUC for predicting an EF increase ≥10% increased to 0.869 from 0.662; p=0.007.

In addition to clinical data and ECG markers of dyssynchrony, our findings demonstrate that scar burden remained an independent marker of worse outcomes based on highly relevant endpoints: death or heart failure and EF improvement. Moreover, our study is the first MRI study to demonstrate the incremental value when added to current CRT clinical selection criteria (presence of LBBB and QRSd).

### Study limitations

There are limitations to consider when interpreting the results of this study. This is an observational, single centre, retrospective study with all the inherent limitations. While we attempted to control for potential confounders, residual confounding cannot be excluded. Also, among patients included, 27 patients did not have an echocardiogram available for review 3 months post-CRT implant, and 26 patients were lost to follow-up before 3 years have passed, which could have biassed our results for these endpoints. Finally, lead characteristics were not available in 17 patients.

## Conclusion

Better identification of patients who would respond to CRT therapy is an important challenge. Scar quantification by CMR appears to independently predict clinical events and LV functional improvement, in both ischaemic and non-ischaemic cardiomyopathy, even when accounting for clinical, electrocardiographic and LV lead characteristics. In addition, scar burden adds incremental value to currently adopted selection criteria for the prediction of CRT response.
