# Supplementary material for: Scar burden is an independent and incremental predictor of cardiac resynchronisation therapy response
Source: Open Heart. 2019 Jul 5;6(2):e001067. doi: 10.1136/openhrt-2019-001067 (PMC6615837; doi:10.1136/openhrt-2019-001067)
Supplement: Supplementary data [file openhrt-2019-001067supp001.docx]

**SUPPLEMENTAL TABLES**

**Supplemental Table 1**: Point and interval estimates of the sensitivity and specificity for various thresholds of total scar percent. Responders are patients who were event-free 3 years after CRT.

| **A patient is considered a good candidate for CRT if:** | **Sensitivity** | | **Specificity** | |
| --- | --- | --- | --- | --- |
|  | **Estimate** | **95% CI** | **Estimate** | **95% CI** |
| **Scar = 0%** | 40% (16/40) | 26%, 55% | 83% (15/18) | 60%, 95% |
| **Scar < 5%** | 68% (27/40) | 52%, 80% | 72% (13/18) | 49%, 88% |
| **Scar < 15%** | 85% (34/40) | 71%, 93% | 67% (12/18) | 44%, 84% |
| **Scar < 33%** | 93% (37/40) | 79%, 98% | 56% (10/18) | 34%, 75% |

Abbreviations: CI = confidence interval, CRT = cardiac resynchronization therapy.

**Supplemental Table 2:** Point and interval estimates of the sensitivity and specificity for various thresholds of total scar %. Responders are patients whose ejection fraction increased by at least 10% after CRT.

| **A patient is considered a good candidate for CRT if:** | **Sensitivity** | | **Specificity** | |
| --- | --- | --- | --- | --- |
|  | **Estimate** | **95% CI** | **Estimate** | **95% CI** |
| **Scar = 0%** | 43% (13/30) | 27%, 61% | 81% (22/27) | 63%, 92% |
| **Scar < 5%** | 77% (23/30) | 59%, 88% | 70% (19/27) | 51%, 84% |
| **Scar < 15%** | 97% (29/30) | 82%, 100% | 52% (14/27) | 34%, 69% |
| **Scar < 33%** | 97% (29/30) | 82%, 100% | 26% (7/27/) | 13%, 45% |

Abbreviations as in previous table.
